# Human Trophoblast Progenitor Cells Express and Release Angiogenic Factors

**DOI:** 10.22088/IJMCM.BUMS.7.4.203

**Published:** 2019-01-28

**Authors:** Muge Molbay, Dijle Kipmen-Korgun, Gizem Korkmaz, Murat Ozekinci, Emin Turkay Korgun

**Affiliations:** 1 *Department of Histology and Embryology, Medical Faculty, Akdeniz University, Antalya, Turkey.*; 2 *Department of Biochemistry, Medical Faculty, Akdeniz University, Antalya, Turkey.*; 3 *Department of Obstetrics and Gynecology, Medical Faculty, Akdeniz University, Antalya, Turkey.*

**Keywords:** Trophoblast progenitor cells, angiogenic factors, CDX2, EOMES

## Abstract

Trophoblast stem cells develop from polar trophectoderm and give rise to the cells that generate the placenta. Trophoblast cells are responsible for the uterine invasion and vascular remodeling during the implantation of the embryo. However this knowledge is not yet to be confirmed for trophoblast progenitor cells (TPCs). In this study, we aimed to demonstrate that human TPCs (hTPCs) express and release angiogenic factors for the first time. TPCs were isolated from the term placenta. Then immunophenotyping was performed by FACS method by analyzing caudal type homeobox 2 (CDX2) and eomesodermin (EOMES). Immunofluorescence staining of CDX2 and EOMES was performed on these cells. Lastly, angiogenesis-related proteins were detected by western blot and ELISA assays. The isolated cells were positive for trophoblast stem cell markers CDX2 and EOMES in 92.5% and 92.7% of cells, respectively showing the characteristics of TPCs. The investigation of vascular endothelial growth factor (VEGF), vascular endothelial growth factor receptor 1 (VEGFR1), and vascular endothelial growth factor receptor 2 (VEGFR2) at protein and mRNA level in comparison with human umbilical vein endothelial cells (HUVECs), revealed that human TPCs (hTPCs) have higher levels of *VEGF* and *VEGFR1* transcripts. Additionally soluble forms of VEGF and VEGFR1 were detected in supernatants of hTPCs. With this information, TPCs seem to be promising for regenerative cell therapies by increasing angiogenesis.

The placenta is a peerless organ that received limited attention in regenerative medicine. Due to being discarded immediately after birth, the placenta is an easily accessible organ, and a good candidate as a source for regenerative medicine because of a high level of stem cells content. Amnion membrane and chorion are rich stem cells source (1). Besides, stem cells isolation from the amniotic fluid is also possible (1). Trophoblast cells are specialized cells in the placenta that mediate the interactions between the fetüs and mother at the feto-maternal interface. The structural properties of these trophoblast cells, and their ability to modulate vascular, endocrine and immunological processes, facilitate and optimize the exchange of metabolites. Importantly, all of these specialized trophoblast cells can be derived from self-renewing, multipotent cells referred to as trophoblast stem cells. Trophoblast stem cells first isolated from mouse blastocytes (2), and from human placenta (3). Trophoblast stem cells are the pioneers of the differentiated cells in the placenta, originated from polar trophectoderm or extraembryonic ectoderm of blastocyte (2). Trophoblast stem cells primarily express caudal type homeobox 2 (*CDX2*), TEA domain transcription factor 4 (*TEAD4*), Eomes, sex determining region Y-box 2 (*SOX2*), GATA binding protein 3 (*GATA3*), transcription factor AP-2 gamma (*TCFAP2c*), SWI/SNF related matrix associated actin dependent regulator of chromatin subfamily a member 4 (*SMARCA4*), ETS protooncogene 2 (*ETS2*), E74 like ETS transcription factor 5 (*ELF5*), estrogen-related receptor beta (*ESRRB*) genes (4, 5). Today, CDX2 and eomes transcription factors are mainly accepted as the trophoblast stem cell markers (6, 7). In addition to these factors, fibroblast growth factor receptor 2 (FGFR2) and bone morphogenetic protein 4 (BMP4) are other known trophoblast stem cell markers (4). In 2016, Genbacev introduced pieces of evidence that integrin alpha 4 (ITGA4) was the highest level expressed factor in trophoblast stem/progenitor cells isolated from term placenta (8). Therefore, a high level of *ITGA4* expression on the surface of the cells, can be used to identify TPCs.

Vascular endothelial growth factor (VEGF) is one of the most important and effective angiogenesis-promoting molecules. It is an endothelial cell-specific mitogen, and initiates signal transduction through two high affinity receptor tyrosine kinases, VEGFR-1 (or FLT-1) and VEGFR-2 (or KDR/FLK-1). Additionally, these two receptors exist also as soluble forms. Soluble form of VEGFR 1 (sVGFR1) is an antagonist of VEGF action, and decreases the level of free VEGF through strong binding to it (9). In contrast to the action of sVEGFR1, sVEGFR2 does not efficiently antagonize the binding of VEGF and does not inhibit vascular endothelial growth factor A (VEGFA)-induced mitogenesis. Instead, sVEGFR2 contains the VEGF-C binding site and traps VEGF-C, and disables its binding and activation of VEGFR3, thus inhibiting lymphangiogenesis (10).

Although little is known about trophoblast stem and progenitor cells, mesenchymal stem cells (MSCs) are considerably effective for therapeutic angiogenesis in ischemia animal models as well as clinical vascular diseases (11). Previously, many studies revealed that both adult bone marrow MSCs (BMSCs) and adipose tissue-derived MSCs (AMSCs) could induce therapeutic angiogenesis (12). In addition to this wide variety of cell source, umbilical cord-derived MSCs (UMSCs) and placental chorionic villi-derived MSCs (PMSCs) have also been reported to display angiogenic activity *in vitro* and *in vivo* (13).Additionally, human placenta-derived MSC-like cells were shown to be present in blood flow, and promote collateral vessel formation in the injured limb upon increased M2-like macrophages accumulation in ischemic tissue. They enhance angiogenesis through an immuno-modulatory mechanism involving T cell-dependent reprogramming of macrophage differentiation toward M2-like phenotype (14). Since there are many different MSC sources, fetal and maternal originated placental MSCs have also been compared. Up to date, it is not clear whether hTPCs isolated from term placenta express any of these angiogenic factors. The aim of this study was to examine whether they express and release VEGF, KDR and FLT-1 for the first time. With this information, new horizons in the therapeutic use of the human term placenta derived could be discovered.

## Materials and Methods

**Isolation of human trophoblast progenitor cells (hTPCs) from term placenta**

Human term placentas of normal pregnancies

(range 38–42 weeks, n= 6) were obtained after spontaneous delivery or caesarean section with informed consent. Approval of the Ethic Committee of the Medical University of Akdeniz was granted. Isolation of hTPCs was performed according to the protocol of Genbacev et al. (8) using enzymatic treatment of the chorion of the placenta with collagenase A, DNase, trypsin and hyaluronidase (all from Sigma, Saint Louis, MO, USA) after manual separation of the chorion. Subsequently, cells were sorted by using magnetic-activated cell sorting (MACS) (Invitrogen, Carlsbad, CA, USA) positively for integrin α 4 and negatively for major histocompatibility complex, class I, A ,B, C (HLA A, B and C). hTPC were cultured in DMEM F12 (Gibco, Invitrogen, Paisley, UK) supplemented with 10 % FBS ( Hyclone, Little Chalfont, UK) and FGF4 and heparin (both Sigma, Saint Louis, MO, USA).

**Immunophenotyping of cells**

The marker phenotype of these hTPCs was analyzed by flow cytometry for CDX2 and EOMES with a fluorescent-activated cell sorting (FACS) Aria III Cell Sorter flow cytometry and the CellQuest software (BD Biosciences, Franklin Lakes, NJ, USA). The cells were also stained by immunofluorescence for these markers for further confirmation. Briefly, cells were inoculated on chamber slides, and the day after they were washed and air dried. Then, hTPCs were fixed with 1:1 concentration acetone: methanol mixture. Slides were incubated with primer antibodies that were rabbit monoclonal Cdx2 (1:100, Cell Signaling, Danvers, MA, USA), rabbit polyclonal Eomes (1:100 dilution, Allele, San Diego, CA, USA) overnight at 4 °C. Rabbit IgG secondary antibody Alexa Flour 20 mg/ml (1:250, Invitrogen, Carlsbad, CA, USA) was used for labeling. Counterstaining was performed by using a mounting medium with DAPI (Vector, Burlingame, CA, USA).

**Sodium dodecyl sulfate (SDS) polyacrylamide gel electrophoresis and western blotting**

hTPCs scraped from flasks using Laemmli buffer (Sigma, Saint Louis, MO, USA) were prepared and total cellular protein was separated by SDS polyacrylamide gel electrophoresis. HUVEC cells, which are known to express these factors, were used as a positive control. Briefly, membranes were incubated with VEGF (1:500, S.Cruz; Dallas, Tx, USA), VEGFR1 (1:1000, Abcam; Cambridge, UK), VEGFR2 (1:500, Abcam; Cambridge, UK), and beta-actin (1:5000, Abcam; Cambridge, UK) antibodies at +4 °C overnight. After washing with Tris buffered saline with Tween 20 (TBS-T; 0.05 M Tris, 0.15 M NaCl, 0.001% Tween 20), membranes were incubated for 2 h at room temperature (RT) with horseradish peroxidase (HRP) conjugated goat anti-rabbit IgG (BioRad; Hercules, CA, USA) and goat anti-mouse IgG (BioRad; Hercules,CA, USA) antibodies. After washing with TBS-T, antibodies were detected by chemiluminescence-based SuperSignal CL HRP Substrate System (Pierce; Waltham, MA, USA). Membranes were exposed to hyperfilm (Amersham; Pittsburgh, PA), which was subsequently analyzed by using Alpha DigiDoc 1000 gel documentation unit (Alpha Innotech Corporation, CA, USA). All results were normalized to beta actin.

**RNA isolation and cDNA synthesis**

Total RNA was isolated from hTPCs scraped from flasks using Trizol Reagent (Invitrogen; Carlsbad, CA, USA) according to the manufac-turer’s instructions. HUVEC cells, which are known to express these factors were used as positive control. RNA pellets were dissolved in RNase- free water and quantified using UV spectrophotometer. Preparation of cDNA was carried out from 2 μg of total RNA using the SuperScript III First- Strand Synthesis System (Invitrogen; Carlsbad, CA, USA) according to manufacturer’s instructions.

**Quantitative Real-Time (q)-PCR analysis**

The expression of *VEGF*, *VEGFR1*, *VEGFR2*, and β-actin was determined by q-PCR using QuantiFast SYBR Green PCR Kit (Qiagen; Germantown, MD). 12,5 μl of SYBR Green (2x) Master Mix was combined with 8.5 μl of nuclease-free water, 10 μM forward/ reverse primers (1 μl each) and 2 μl of cDNA in the PCR strip tubes. Amplification used 35 cycles of PCR in Applied Biosystems StepONEplus Real-Time PCR System, with the following program: initial denaturation at 95 °C for 10 min, followed by 35 cycles at 95 °C for 15 s, annealing for 30 s and extension at 72 °C for 30 s with a final melting curve at 95 °C 1 min and 55 °C 1 min. All samples were run in duplicate and the average was used for each sample. Expression of the target mRNAs was normalized to β-actin levels and the 2^−ΔΔCt^ values were used to evaluate relative expression levels. Primer sequences are as follows:

*vegf* f:5'-GCAGAATCATCACGAAGTGG-3',r:5'-CTGCATGGTGATGTTGGACT-3', 

*vegfr1* f:5'-GCAAGATTCACCTATG-3', r:5'-CGAGGTTCCTTGAAVAGTGA-3', 

*vegfr2* f:5'-GCATCGAGCTCTCATGTCTG-3, r:5'-CTGGCTACTGGTGATGCTGT-3', 

*beta actin* f:5'-CATGAAGATCCTGACC GAGC-3', r:5'-CAGACAGCACTGTGTTGGCA-3'

**Enzyme linked immunosorbent assay (ELISA) analyzes**

Commercial ELISA kits were used per manufacturer’s instructions. Soluble VEGF-A (sVEGF-A), sVEGFR1 and sVEGFR2 (all, R&D Systems; Minneapolis, MA, USA) concentrations were determined in supernatants of the hTPCs.

**Fig. 1:**
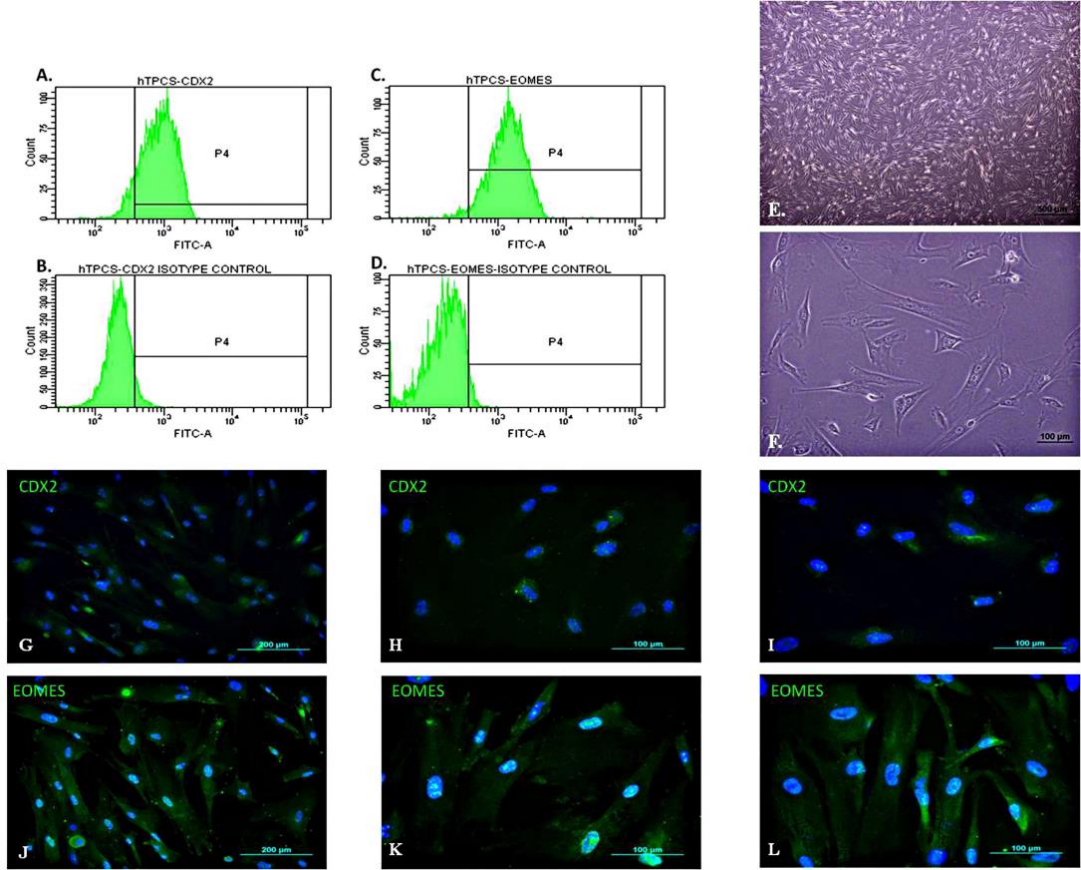
**hTPC characterization.** Flow cytometry analysis of hAMSCs for CDX2 and isotype control (**a, b**) and EOMES and its isotype control (**c, d**). The hTPCs expressed CDX2 and EOMES in 92.5% and 92.7% of cells, respectively. Phase contrast microscopic images of hTPCs in 4x (**e**) and 20x magnifications (**f**). Immunoflourescent characterization of hTPCs with CDX2 (**g, h, i**) and EOMES (**j, k, l**). Magnifications are 10x, 20x and 40x, respectively

**Fig. 2:**
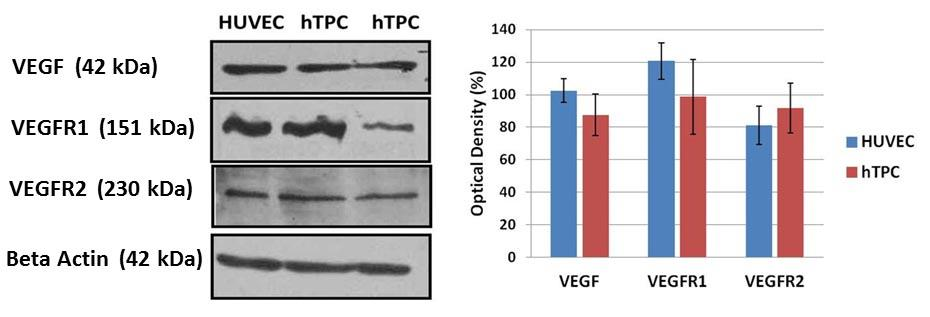
**Western blot results of VEGF, VEGFR1 and VEGFR2 proteins in the HUVEC and hTPCs.** Proteins levels between HUVEC and hTPCs were not statistically significant. All results were normalized to beta actin

**Fig. 3:**
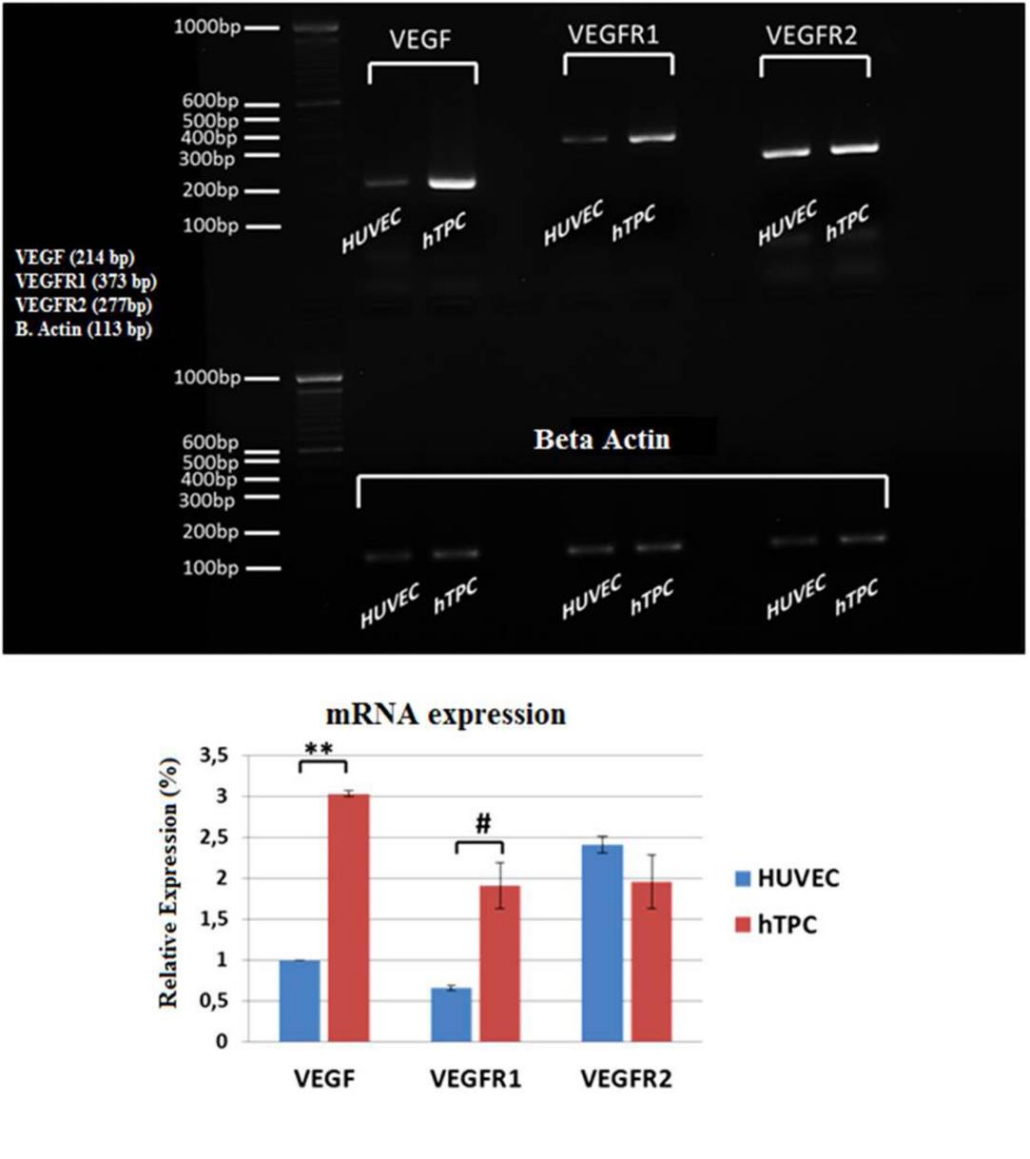
**mRNA levels of angiogenic factors in the HUVEC and hTPCs.** *VEGF* and *VEGFR1 *mRNA were significantly increased in hTPCs (** P ≤ 0.01) in comparison with HUVECs (# P ≤ 0.05). *VEGFR2* mRNA levels were similar between all groups. All results were normalized to beta actin

**Fig. 4:**
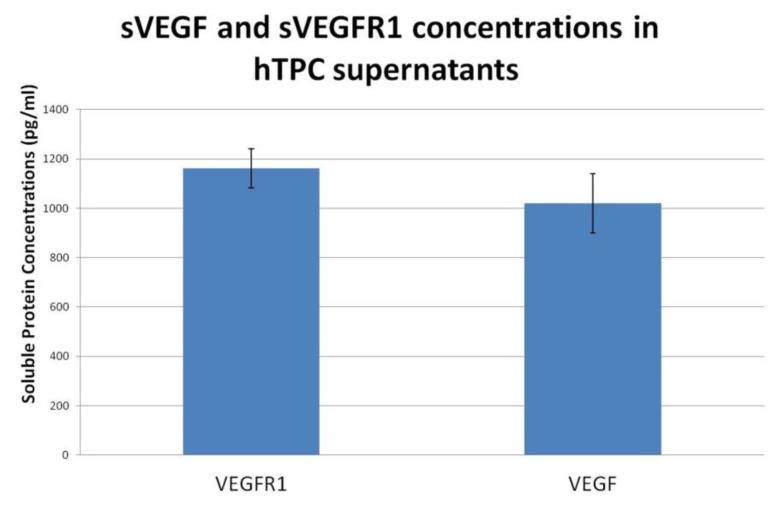
Soluble forms of VEGF and VEGFR1 proteins in supernatants of hTPCs

Briefly, the appropriate amount of samples were pipetted into pre- coated 96 well plates and incubated for 2 h at RT. After washing, 200 µl HRP-conjugated antibody was added and incubated for 2 h. A 200 µl aliquot of substrate solution was then added and incubated in the dark for 30 min. After stopping the reaction, the plate was read at 450 nm using a spectrophotometer.

**Statistical analyzes**

Data were expressed as mean ±SEM. One way ANOVA test was used to compare the means of multiple groups. One Way Anova was performed using the SigmaStat 3.5 software. A value of p ≤0.05 was considered as statistically significant. Western blot band densities were measured with the Alpha DigiDoc 1000 gel documentation unit. Densitometric data obtained from western blot and 2^-ΔΔCt^ values obtained from Q-PCR analysis were subjected to statistical analysis using the SigmaStat 3.5 software. One way Anova was performed and p≤0.05 was considered as statistically significant. For ELISA analysis, protein concentrations were evaluated and p≤0.05 was considered as statistically significant.

## Results

**Isolated cells bear the characteristics of hTPCs**

hTPCs showed fibroblast-like phenotype at passage 3, and were characterized with cell surface markers by flow cytometry analysis of CDX2 and EOMES. The percentages of cell surface markers were 92.5% for CDX2 (Fig.1a, b) and 92.7% for EOMES (Fig.1c, d). Morphologically, hTPCs showed a spindle or triangular phenotype, and adhered to plastic in the second passage (Fig.1e and f). Immunofluorescence staining of the cells provided further evaluation. It has been shown that isolated cells expressed trophoblast cell markers CDX2 (Fig.1g, h and i) and EOMES (Fig.1j, k and l).

**hTPCs express and release angiogenic factors**

Immunoblotting results prove that hTPCs express VEGF, VEGFR1, and VEGFR2. In comparison to positive control HUVEC, hTPCs have lower VEGF and VEGFR1 protein level and elevated VEGFR2 protein level (Fig. 2).

Q-PCR analysis confirmed the presence of mRNAs of all three factors; VEGF, VEGFR1 and VEGFR2 (Fig.3). In comparison with HUVECs, hTPCs have significantly higher *VEGF* (P≤0.01) and *VEGFR1* (P ≤0.05) mRNA levels.

Soluble forms of VEGF and VEGFR1 were present in supernatants of hTPCs (Fig. 4). ELISA study demonstrated that the high level of sVEGFR1 was released from hTPCs to media. The soluble form of VEGFR2 in hTPC supernatants could not be detected.

## Discussion

Normal vascularization of the placenta starts around day 21 post conception, and it is a consequence of *de novo* vessel formation from pluripotent precursor cells in the mesenchymal core of the villi. Previously, immunocharacterization of placental vascularization during early pregnancy (days 22–48 post conception) under electron and light microscopy revealed that *VEGF* is strongly expressed in villous cytotrophoblasts and later Hofbauer cells, and *VEGFR1* and *VEGFR2* are expressed on vasculogenic and angiogenic precursor cells (15).

Many previous studies revealed that angiogenic factor *VEGF* is expressed by many types of MSCs. Considering the angiogenic activity of the placenta, placenta-derived stem cells independent from the source appear to express this factor, either. UMSCs and PMSCs have also been reported to display angiogenic activity *in vitro* and *in vivo* (13). As previously mentioned, PMSCs of fetal and maternal origins demonstrate different therapeutic potentials (14). This study claims that cells derived from the fetal part of the placenta demonstrate significantly high potentials in promoting angiogenesis *in vitro*, and increasing immunosuppressive function *in vivo*. Besides, in rat focal cerebral ischemia model our group performed, infarct size of ischemic rats was found to be smaller in hTPCs transplanted rats upon 2% TTC (2,3,5- Triphenyltetrazolium) treatment (unpublished data). Since hTPCs are isolated directly from the chorion, the fetal part of placenta, this may point additional therapeutic effect they have due to their niche. Consisting with our results, another additional information about their niche is that conditioned medium of chorion-derived mesenchymal stromal cells contains important amounts of proangiogenic factors which are secreted by hAMCs, including VEGF, bFGF, IGF-1, thrombopoietin, PDGF-BB, angiogenin, and VEGF-D (20). Furthermore, because trophoblast cells are the main cells in the placenta, and hTPCs are their ancestors and are the frontiers in placental formation, it seems logical to expect greater therapeutic potential. Though, comparison studies between these cells still are required to be performed.

Despite the fact that VEGFR1 is mainly known as the decoy receptor, and as an inhibitor through its soluble form, there are many findings suggesting that sVEGFR1 may contribute to angiogenesis. Although the binding affinity of VEGFR1 to VEGF is high, it has poor kinase activity. In contrast, VEGFR2 can not bind PLGF, and binds to VEGFA with a lower affinity. However, thanks to high receptor kinase activity, signal transduction results in eminent proliferation and chemotactic response. However in placenta, VEGF could induce the formation of not only VEGFR1/VEGFR1 receptor homodimers but also, on the basis of soluble receptor complexes, VEGFR1/VEGFR2 receptor heterodimers allow the use of the full receptor repertoire. Moreover, VEGF/PlGF ligand heterodimer might be expected to bind not only VEGFR1 homodimers but also VEGFR1/VEGFR2 receptor heterodimers. sVEGFR1 might participate in VEGF-induced mitogenesis by transactivation of the VEGFR2 tyrosine kinase through receptor heterodimer formation on the cell surface. The weak complexes that sVEGFR1 form with the extracellular region of VEGFR2 that appear to be stabilized by VEGF, supports this theory (17). Furthermore, functional VEGFR1 receptor expression by human trophoblast cells for VEGF triggering the synthesis and release of nitric oxide (NO) by the activation of constitutive NO synthase (cNOS) was reported previously (18). Especially for trophoblast cells, whose microenvironment is rich in PlGF, this might be another mechanism to induce angiogenesis. Our results agree with this hypothesis as a large amount of VEGFR1 expression is detected on both protein and mRNA level. Additionally, tremendous levels of soluble VEGFR1 were identified in hTPCs supernatants upon ELISA tests. Furthermore, cNOS activation through VEGFR1 may result in further angiogenic activation in addition to VEGFR2 activity. For recruitment therapies targeting to enhance angiogenesis, this property of hTPCs may be invaluable. On the other hand, sVEGFR2 did not exist in the supernatants. This may point out that hTPCs do not have the anti-lymphangiogenic effect.

To sum up, to date, villous trophoblasts proved themselves as angiogenic promoters through their secretions (19). With the information from this study, it is seen that this angiogenic provocative ability may be inherited from their stem cell ancestor through their progenitor predecessor ending up to villous cytotrophoblast.

Trophoblast cells are immune privileged cells for fetus inhibiting T cell and natural killer cell (NK) responses (20), thus, they are advantageous for clinical use for the fetus. Thanks to their ability to differentiate into other cells and their immune suppressive ability, they are very important for cell and gene therapy treatments.

There is much evidence that placental cells have *in vivo* regenerative potential. During pregnancy, fetal cell microchimerism is observed. Trophoblast cells join the maternal circulation and they can be transplanted into damaged organs by migrating through inflammation. It was also reported that pregnancy cardiomyopathy has the highest odds of spontaneously recovery of about 50% (21). Researches performed by Kara et al. addressed the reason underneath this extraordinary result. In the myocardiac infarct mouse model, they displayed fetal cells traffic to injured maternal myocardium, and these cells underwent cardiac differentiation in maternal hearts**. **These cells were CDX2 and EOMES positive, therefore it is highly possible that they were TSCs (22).

hTPCs appear to be potential alternative pluripotent cells for the treatment of Parkison’s disease and other neurodegenerative diseases. It was shown that retinoic acid-induced TPCs to differentiate into dopaminergic neurons and these cells regenerated nigrostriatal pathway and reduced motor neuron deficiency in a functional manner for both acute and chronic Parkison’s disease model rats (23).

In the present study, we displayed that TPCs may induce angiogenesis in transplantation era which enhances the recovery process. Therefore, they seem to be strong candidates for recruitment therapy. Many question marks still hang on TPCs topic so this topic, needs further researches.
